# Different gut microbiome dysbioses impact the evolutionary fate of hypervirulent *Klebsiella pneumoniae*

**DOI:** 10.1002/imt2.70175

**Published:** 2026-09-23

**Authors:** Carey Lim, Jialing Zheng, Guoxiang Cheam, Melvin Yong, Hanrong Chen, Andrew Ting, Yahua Chen, Qingqing Wang, Niranjan Nagarajan, Yunn‐Hwen Gan

**Affiliations:** ^1^ Infectious Diseases Translational Research Programme, Yong Loo Lin School of Medicine National University of Singapore Singapore Republic of Singapore; ^2^ Department of Biochemistry, Yong Loo Lin School of Medicine National University of Singapore Singapore Republic of Singapore; ^3^ Institute of Immunology Zhejiang University School of Medicine, Hangzhou, and Liangzhu Laboratory Hangzhou China; ^4^ Genome Institute of Singapore (GIS), Agency for Science Technology and Research (A*STAR) Singapore Republic of Singapore

**Keywords:** antibiotic, capsule, colonization, gut microbiome, hypermucoviscosity, *Klebsiella pneumoniae*, short‐chain fatty acids

## Abstract

The intestinal microbiota acts as a barrier against pathogen colonization, yet how specific ecological perturbations shape the evolutionary fate of invading pathogens remains unclear. Here, we show that the mode of gut perturbation and type of dysbiosis determine whether hypervirulent *Klebsiella pneumoniae* (hv*Kp*) persist or clear from the intestines. Transient ampicillin exposure creates a permissive, low‐diversity intestinal niche dominated by facultative anaerobes, which supports long‐term, high‐density hv*Kp* colonization. The dysbiotic state creates an altered redox environment to favor these oxygen‐using bacteria and is associated with the rapid emergence of hypomucoid variants. This occurs via contractions in the poly‐T tract in the promoter of the *rmpA* regulator that controls capsule hypermucoviscosity, as well as mutations in the poly‐G tract within the coding sequence. This evolutionary trajectory reflects a virulence‐persistence trade‐off in high‐nutrient, low‐competition environment. Conversely, osmotic perturbation via laxative treatment elicits only a transient window of colonization‐susceptibility that is rapidly closed by the recovery of a *Bacteroides*‐enriched community that is consistent with their ability to actively suppress pathogen colonization via short‐chain fatty acids (SCFAs). Furthermore, SCFAs such as butyrate are more effective in inhibiting hypomucoid *rmpA* variants relative to wild‐type bacteria, providing a possible explanation for how an osmotically perturbed gut microbiome constrains the expansion of these mutants. These findings provide insights into how transient antibiotic exposure could potentially drive long‐term intestinal pathobiont colonization while laxative‐induced osmotic perturbations do not rewire the energy and metabolic landscape of the gut microbiome, enabling rapid recovery and colonization resistance despite an initial plunge in diversity. Our work reveals that the specific mode of perturbation affects the trajectory for pathogen adaptation, and highlights the potential role of commensal competition in constraining pathogen evolution.

## Highlights

- Different transient physiological perturbations of the gut, such as ampicillin or osmotic laxative exposure, result in hypervirulent *K. pneumoniae* colonization persistence or decolonization respectively.
- Ampicillin exposure favors the emergence of hypomucoid variants through promoter poly‐T and coding sequence poly‐G contraction/expansion in the *rmpA* gene.
- Short‐chain fatty acids produced by gut microbiome from laxative‐exposed gut microbiome may help constrain the expansion of *rmpA* hypomucoid variants.

## Graphical abstract

Transient exposure to ampicillin results in restructuring of microbiota to favor oxygen‐tolerant bacteria, whereas osmotic laxative perturbation preserves Bacteriodota. Ampicillin‐ but not laxative‐exposed environment favors establishment of *rmpA* phase variants that lost hypermucoviscosity but short‐chain fatty acids present in the laxative‐exposed gut may constrain the establishment of these phase variants.

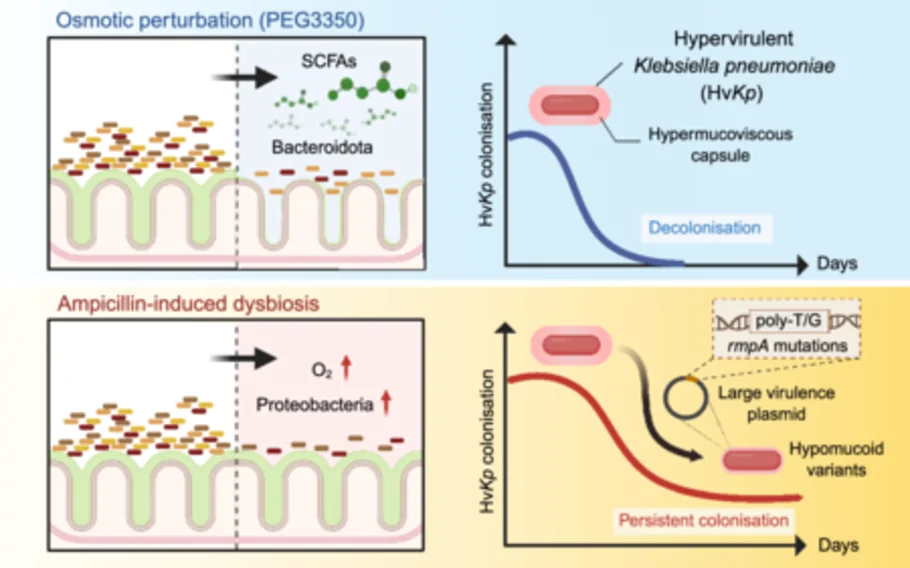

## INTRODUCTION

The healthy intact gut microbiota functions as a primary defence system, exerting colonization resistance against pathobionts through nutrient competition, niche occupation, and host immune modulation at the intestinal barrier [1, 2, 3]. However, this protective barrier is fragile. Disruptions to the resident microbiota can deplete protective commensals and liberate resources, generating a dysbiotic state that permits the bloom of opportunistic pathobionts such as hypervirulent *Klebsiella pneumoniae* (hv*Kp*) [4, 5].

Gut colonization by *K. pneumoniae* is a critical determinant of clinical outcome, serving as an important reservoir for dissemination to the bloodstream that can lead to metastatic infections [6, 7, 8]. A previous study involving 1765 hospitalized patients demonstrated that intestinal colonization with *K. pneumoniae* was strongly associated with the development of subsequent infections [9]. Furthermore, hv*Kp* is believed to cause monomicrobial liver abscess via transit from the gut through the hepatic portal vein to the liver [10]. The gut is considered an important reservoir where Enterobacterales can acquire multidrug‐resistance plasmids [11] and undergo adaptive diversification prior to systemic spread. While the specific bacterial fitness factors required for gut residency have been extensively characterized [12], the extent to which distinct host and microbiota‐derived macroenvironments dictate these colonization dynamics remains poorly understood. Furthermore, how bacteria evolve and adapt to distinctive host environments that could alter clinical outcomes is not well understood. Previous studies have documented the emergence of capsule and hypermucoviscosity (HMV) loss mutants, particularly in urinary isolates. Song et al. reported the emergence of hypomucoid variants from a CR‐hv*Kp* strain in the urinary tract of a patient because of capsule loss as well as reduced HMV due to IS*Kpn26* insertion or deletion [13]. CR‐*Kp* urinary isolates belonging to ST258 have also been documented to have deletions in capsule biosynthetic genes as well as loss of HMV due to mutations in the *wzc* gene [14]. In the gut, an ST258 CR‐*Kp* isolate was shown to become capsule‐null due to insertion sequence disruption of its capsular operon [15].

Current research often relies on antibiotic‐induced dysbiosis models to study pathogen establishment [16, 17]. However, the gut is also frequently subjected to non‐antibiotic physiological disturbances, such as osmotic stress caused by common laxatives that can physically alter the mucosal barrier and restructure the microbiome [18, 19]. It remains unclear whether these physical perturbations create windows of vulnerability analogous to those driven by antimicrobial agents, or impose distinct selective pressures on the invading pathogen, particularly hv*Kp*.

Here, we compare two common physiological but distinct modes of intestinal disturbance (ampicillin‐induced dysbiosis and laxative‐induced osmotic stress) to investigate how divergent gut macroenvironments shape hv*Kp* persistence and evolution. We show that while both treatments disrupt gut homeostasis, they engender fundamentally distinct ecological and evolutionary landscapes. Specifically, antibiotics foster a long‐term, persistent high‐density reservoir conducive to the emergence of HMV‐loss mutants, whereas osmotic stress results in a transient colonization window rapidly closed by the recovery of competitive commensal anaerobes.

## RESULTS

### Distinct physiological triggers result in different colonization outcomes

**Figure 1:**
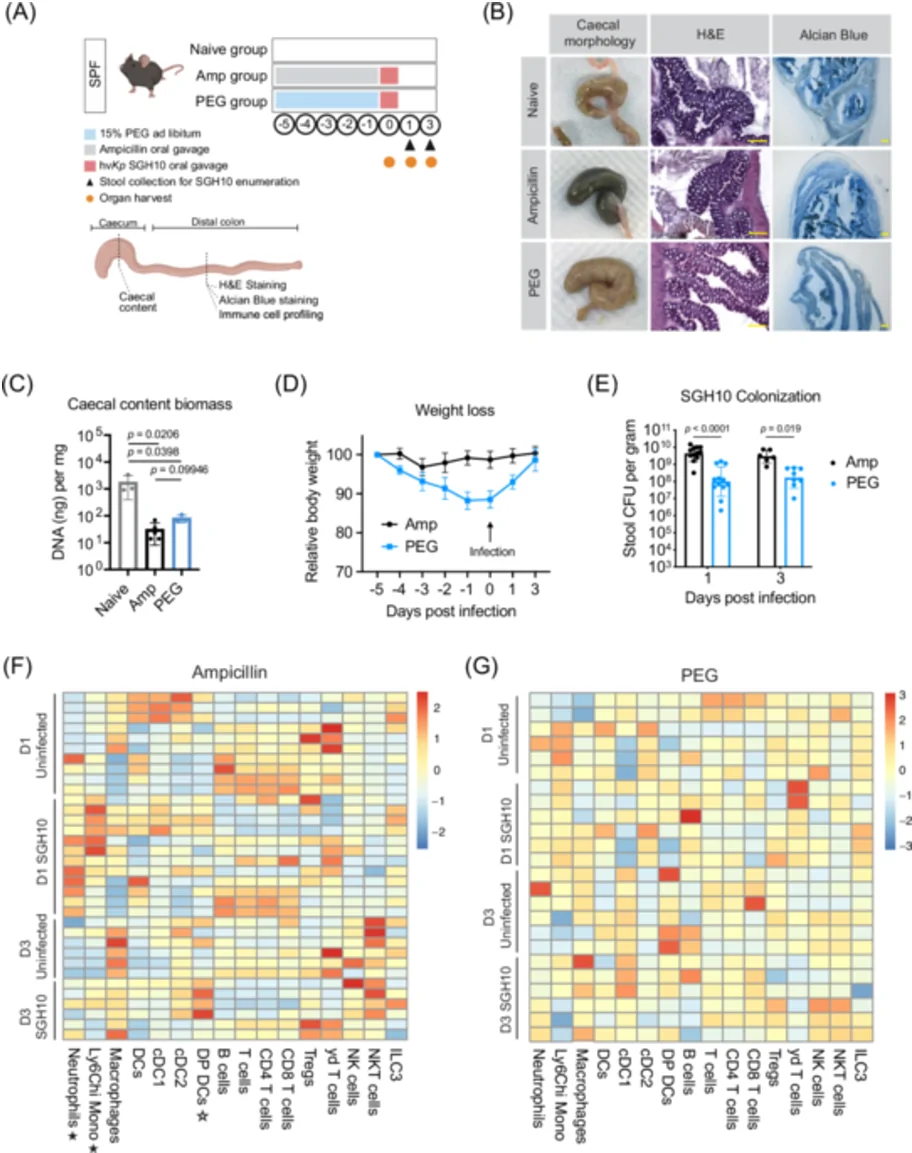
Effects of ampicillin and osmotic laxative (PEG) pre‐treatment on host physiology and *K. pneumoniae* SGH10 colonization dynamics. (A) Schematic overview of ampicillin and osmotic diarrheal pre‐treatment regimens used to establish gut colonization in C57BL/6 mice with hv*Kp* SGH10. (B) Representative phenotypes in naïve, ampicillin‐treated and PEG‐treated mice, including gross morphology of caeca and haematoxylin‐eosin histopathology and alcian blue staining of colon. Scale bars represent 200 μm. (C) Quantification of total biomass in naïve, ampicillin‐treated and PEG‐treated mice, measured as total DNA per mg of dry cecal content. (D) Percentage weight change in mice treated with ampicillin or PEG for 5 days prior to SGH10 challenge (*n* = 8). Arrow indicates the timing of SGH10 introduction by oral gavage. (E) SGH10 colonization loads at 1 and 3 dpi following 5 days of either ampicillin or PEG pre‐treatment. CFU counts were log_10_‐transformed and analyzed by two‐way ANOVA with Sidak's multiple comparison test. (F and G) Heatmaps of Z‐scores for immune cell abundances in colonic lamina propria, expressed as a percentage of total CD45^+^ cells, comparing naïve to ampicillin‐treated mice (left) and naïve to PEG‐treated mice (right) at 1 and 3 dpi (*n* = 6‐12) with uninfected controls. Population changes (% CD45) were analyzed by two‐way ANOVA. A black star denotes statistically significant differences between D1 uninfected and SGH10‐infected mice (Neutrophils, *p* = 0.0008; Ly6C^hi^ monocytes, *p* = 0.0006), while an open star denotes statistically significant differences between D3 uninfected and SGH10‐infected mice (CD103^+^CD11b^+^ DP dendritic cells, *p* = 0.029). Data in (C) were analyzed by one‐way ANOVA. Each point denotes an individual mouse. Data in (C) and (D) are shown as mean ± SD. Data in (E) are shown as geometric mean ± geometric SD.

To dissect how specific gut perturbations influence hv*Kp* establishment, we employed two murine models of intestinal colonization to compare how they influence hv*Kp* colonization: (i) antibiotic‐induced dysbiosis via 5 days of oral ampicillin gavage, and (ii) 5 days of osmotic perturbation using the laxative polyethylene glycol 3350 (PEG) in drinking water, representing a non‐antibiotic perturbation of gut homeostasis (Figure 1A). Both treatments induced significant physiological changes, including cecal enlargement and reduced cecal content biomass compared to naïve controls (Figure 1B,C). However, distinct phenotypic signatures were also observed for each treatment. Ampicillin exposure caused cecal discolouration (Figure 1B) while PEG resulted in a greater increase in cecal content weight (Figure S1A) and reversible weight loss in mice (Figure 1D). Neither treatment appeared to cause large‐scale damage of the intestinal epithelium, but PEG treatment displayed a marked loss of mucus (Figure 1B). Additionally, immune profiling of the colon showed both treatments caused an initial contraction of the myeloid compartment (e.g., macrophages and dendritic cells) but increased lymphocytic cell accumulation in the colon (Figure S1B,C, Figure S6). Following oral challenge with hv*Kp* strain SGH10, the two environments supported vastly different colonization trajectories. Ampicillin treatment facilitated immediate, high‐density colonization (2 logs higher than PEG‐treated mice at Days 1 and 3) (Figure 1E). Furthermore, the two treatments elicited distinct host immune signatures in the colon following SGH10 introduction. One day post‐infection, the ampicillin‐treated group exhibited an increase in neutrophils and inflammatory Ly6C^hi^ monocytes, which largely returned to baseline by Day 3 (Figure 1F). Conversely, PEG treatment did not result in a similarly potent inflammatory infiltration upon colonization (Figure 1G). Notably, only the ampicillin‐treated group displayed transient elevations of inflammatory cytokines such as IL‐6 and TNF‐α 1 day post‐infection (Figure S1D).

**Figure 2:**
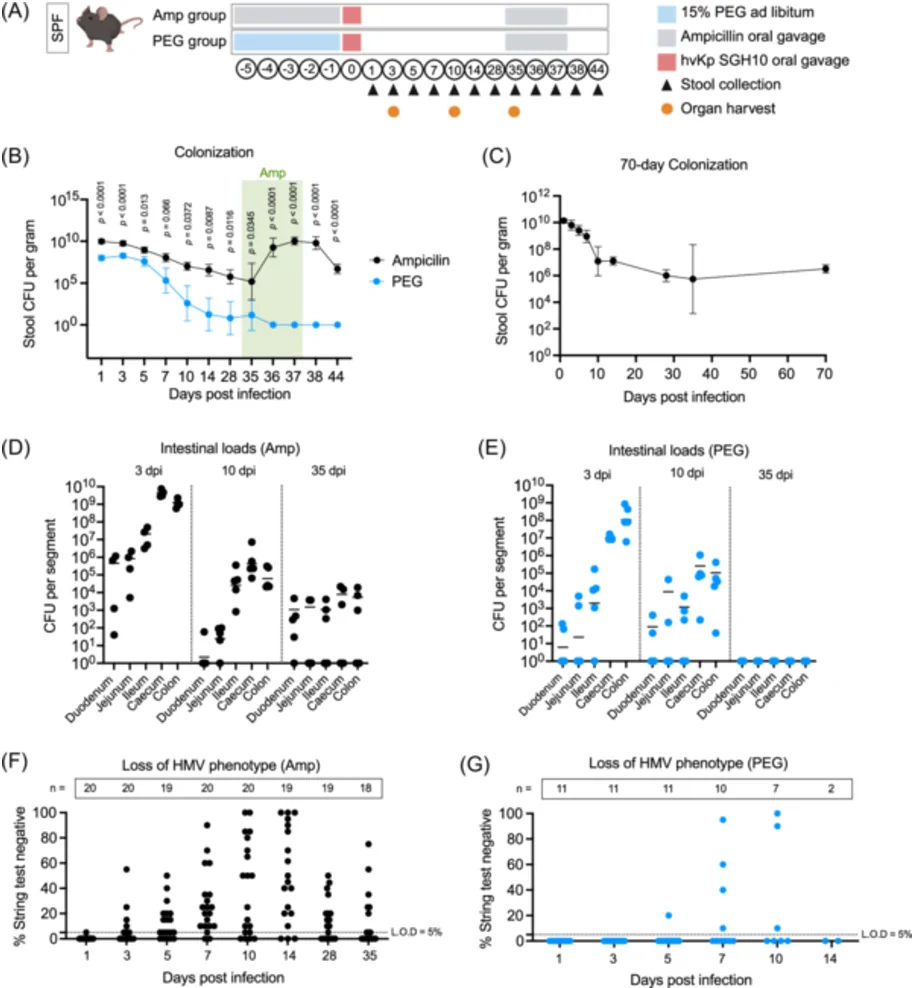
Ampicillin‐induced niche leads to long‐term persistence of SGH10 and selects for loss of HMV. (A) Experimental design for long‐term SGH10 colonization in ampicillin‐ or PEG‐treated mice. (B) Longitudinal SGH10 colonization over 35 days in ampicillin‐ and PEG‐treated mice, followed by re‐exposure to ampicillin for 3 days (*n* = 6–8). Green bar indicates the period of ampicillin reintroduction. Where no colony was enumerated at the limit of detection, data were plotted as 1 on the log axis. CFU counts were log_10_‐transformed and analyzed by two‐way mixed‐effects model, with Sidak's multiple‐comparisons test. (C) Extended SGH10 colonization trajectory in ampicillin‐treated mice monitored for 70 days (*n* = 7). (D, E) Colonization loads in each part of the lower digestive tract at 3, 10 and 35 dpi in ampicillin‐ and PEG‐treated mice. (F, G) Proportion of string test negative colonies (out of 20 randomly selected colonies per mouse) isolated from stools of ampicillin (left)‐ and PEG‐treated mice (right) over 35 days of colonization (limit of detection = 5%). The number of animals per time point is represented above the graph. Sampling for PEG‐treated mice ended on 14 dpi due to SGH10 decolonization. Each point denotes an individual mouse. Data in (B–E) are shown as geometric mean ± geometric SD. Timepoints on the *x*‐axis of (B) are not plotted to scale.

To examine the colonization trajectories resulting from the two models of perturbation, we examined hv*Kp* colonization up to 35 days post‐infection (Figure 2A). Mice in the ampicillin‐treated group maintained higher loads compared to the PEG‐treated group throughout the experiment, with a load of ~10^6^ CFU at day 35 (Figure 2B). Conversely, PEG treatment created only a transient window for colonization. Bacterial loads declined rapidly and most mice achieved complete decolonization between Day 14 and 28 (Figure 2B). To confirm clearance, we re‐introduced ampicillin on Day 35 (Figure 2A). While the ampicillin‐treated group exhibited a bloom reaching original carrying capacities, the PEG‐treated group showed no resurgence of hv*Kp*, confirming successful elimination of SGH10 from the gut (Figure 2B). In a separate experiment, we examined the colonization of ampicillin‐treated animals for up to 70 days, which demonstrated persistent and stable gut colonization between Day 35 and Day 70 (Figure 2C). To validate that fecal output served as a reliable proxy for intestinal colonization loads, we assessed bacterial loads across the gastrointestinal tract. In both groups, the highest bacterial burdens were localized to the cecum and colon (Figure 2D,E), indicating the large intestine as the primary colonization niche without differential redistribution to the small intestine. Notably, while the ampicillin group exhibited sustained persistence, the high‐density reservoir in the caecum and colon eventually contracted, dropping by Day 35 to levels comparable to those in the small intestine.

During the enumeration of bacterial loads, we observed the emergence of colonies that had lost the characteristic HMV phenotype of hv*Kp* (Figure S2A). We examined 20 colonies per mouse to assess the proportion of HMV loss over 35 days. In the ampicillin‐treated group, colonies showed significantly greater variation and higher HMV loss from Day 3, with more mice carrying HMV loss mutants at Days 10 and 14 (Figure 2F,G). Sampling of the PEG‐treated group stopped on Day 14, as mice either had low loads or were decolonized by then.

### Antibiotic‐induced niche selects for loss of HMV

**Figure 3:**
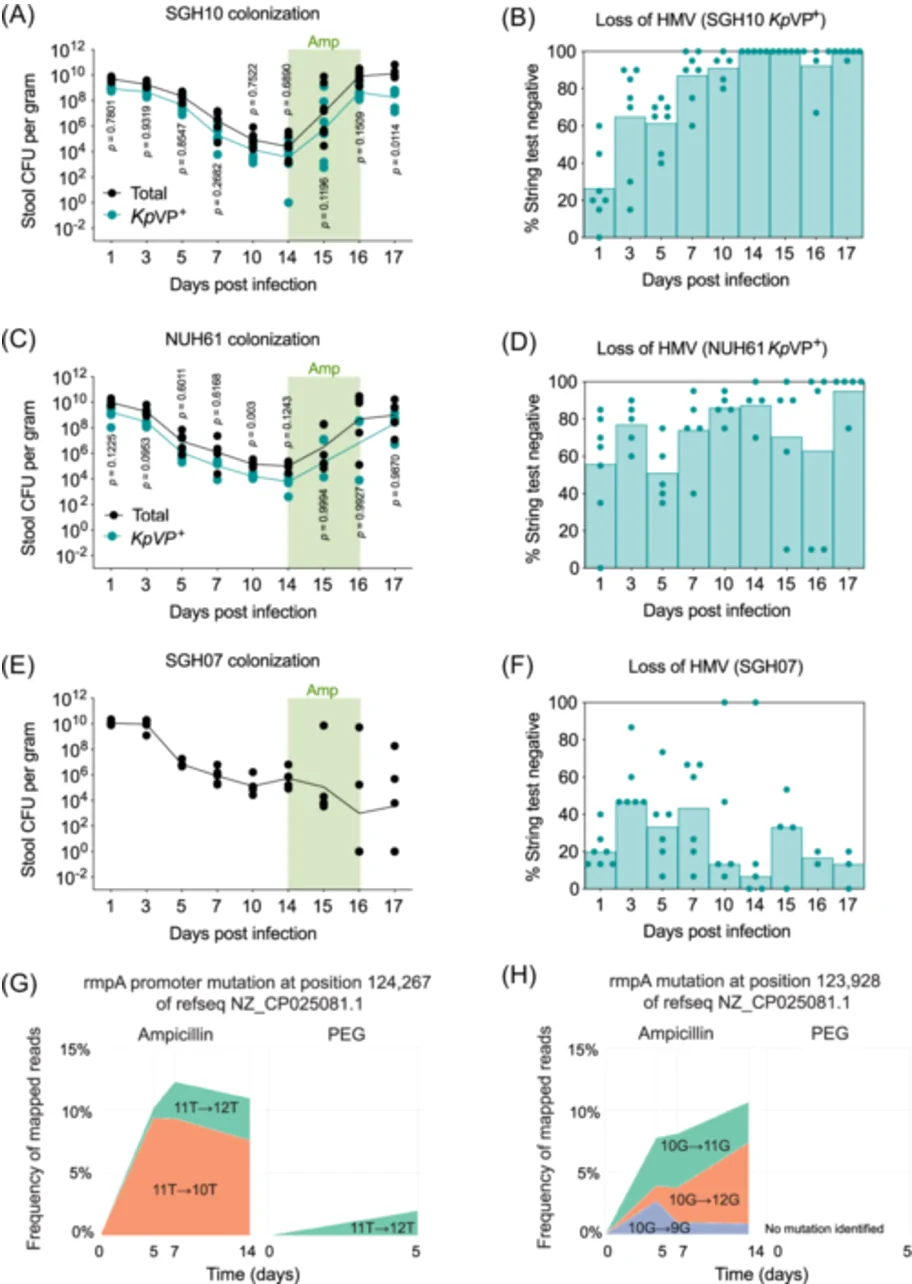
Ampicillin treatment and not PEG treatment results in loss of HMV through *rmpA* mutations and hypervirulent plasmid loss. (A) Colonization kinetics detailing both total and *Kp*VP^+^ SGH10 subpopulation counts over time (*n* = 7 for all timepoints except 17 dpi, where *n* = 6). (B) Percentage of string‐test negative colonies (out of 15 randomly selected per mouse) in colonized mice across different time points. (C) Colonization kinetics detailing both total and *Kp*VP^+^ NUH61 subpopulation counts over time (*n* = 5 for all timepoints except 1 dpi, where *n* = 7). (D) Percentage of string test negative colonies in NUH61‐colonized mice across different time points. (E) Colonization kinetics of SGH07 over time (*n* = 7 at 1dpi, *n* = 6 at 3–7 dpi, and *n* = 5 at 10–17 dpi). (F) Percentage of string test negative colonies in SGH07‐infected mice across different time points. For (A) and (C), CFU counts were log_10_‐transformed and analyzed by two‐way mixed‐effects model, with Sidak's multiple‐comparisons test. Data are shown as geometric means ± geometric SD. Means are represented in bar charts. Each point denotes an individual mouse. (G, H) Stacked area chart illustrating allele frequencies of nucleotide repeat length variations within the *rmpA* promoter region (11 T → 10 T or 12 T; position 124,267 of NZ_CP025081.1) (G) and the *rmpA* coding region (10 G → 9 G, 11 G or 12 G; position 123,928 of NZ_CP025081.1) (H) comparing between ampicillin‐ (left) and PEG‐treated (right) conditions. (*n* = 5–6 per group). Timepoints on the *x*‐axis of (A–F) are not plotted to scale.

Initial sequencing of random non‐mucoid isolates identified two types of mutations: the loss of the large virulence plasmid (*Kp*VP) and mutations in the *rmpA* promoter region (Figure S2B). We then evaluated whether HMV loss was predominantly driven by *Kp*VP loss or by mutations in the *rmpA* locus. Since *Kp*VP encodes tellurite resistance genes, we could determine the proportion of SGH10 that had lost *Kp*VP by plating on tellurite‐supplemented agar (Figure S2C,D). Our data showed that most of the population retained *Kp*VP. While not statistically significant for most timepoints, there was a trend towards an estimated 10% loss of *Kp*VP in the total population that remained relatively constant over time (Figure 3A). We also re‐introduced ampicillin on Days 14–16 to examine whether ampicillin treatment would promote further HMV loss. Among colonies retaining *Kp*VP, we indeed observed high HMV loss (nearly 100%) after ampicillin reintroduction (Figure 3B). The phenomenon of HMV loss during colonization was also conserved across different *K. pneumoniae* lineages, although the frequencies after ampicillin reintroduction varied by strain. Specifically, strain NUH61 (ST86, K2) mirrored SGH10 (ST23, K1), with approximately 10% of the population showing loss of *Kp*VP; within the *Kp*VP‐retaining population, HMV loss frequently exceeded that observed in SGH10 at early timepoints (Figure 3C,D). Strain SGH07 (ST60, K5), which lacks *Kp*VP but encodes *rmp* chromosomally, similarly yielded non‐mucoid variants, although the overall frequency of HMV loss was lower than that observed in the plasmid‐bearing strains (Figure 3E,F).

We then examined HMV loss among *Kp*VP‐retaining SGH10 clones, as this would represent other forms of mutations in the *rmp* operon. To identify the specific mutations driving this phenotypic shift, we performed population‐wide variant calling on the capsule biosynthesis operon (*galF* to *ugd*) and its regulatory genes (*rmpA*, *rmpC*, and *rmpD*). While both treatment groups harbored SGH10 subpopulations with expansions in the *rmpA* promoter poly‐T tract (11 T → 12 T), only ampicillin‐treated mice showed enrichment for *rmpA* promoter contractions (11 T → 10 T; 5–10% allele frequencies) (Figure 3G) and variations in the poly‐G tract of *rmpA* coding sequence (10 G → 9 G, 11 G, or 12 G; combined allele frequency slightly above 10%) (Figure 3H), all of which result in truncated *rmpA* protein. Repeat‐length variants were also observed, albeit less frequently, in *rmpC* (9 G → 8 G) and *rmpD* (8 T → 9 T) in both ampicillin‐ and PEG‐treated groups (Table S1). Mutations in the capsule operon were sporadic. Sequencing of the *rmp* locus in several non‐HMV NUH61 colonies revealed both promoter and coding sequence mutations in *rmpA* as observed in SGH10 (Figure S2E), whereas in SGH07, we isolated a single non‐HMV mutant harboring contraction of the poly‐T tract within the *rmpA* promoter (10 T → 9 T) (Figure S2F). Whole genome sequencing of the entire bacterial lawn from SGH07‐colonized mouse stools at Days 5 and 7 further revealed other *rmp* locus mutants, including frame‐shift mutants (mice 3 and 4) at low frequencies (Figure S2G), consistent with the lower sampling frequency of non‐HMV colonies observed for this strain.

### *RmpA* mutations confer a competitive fitness advantage in the antibiotic‐induced dysbiotic gut

**Figure 4:**
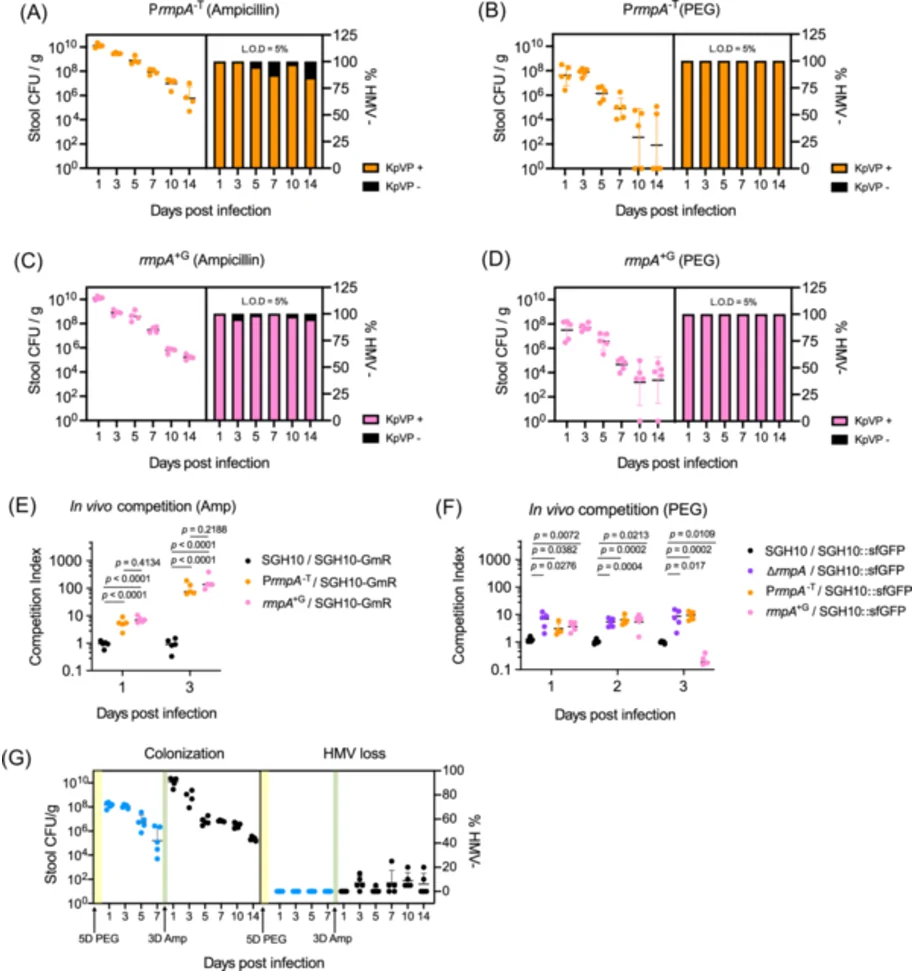
*rmpA* mutations confer some fitness advantage in the ampicillin‐treated dysbiotic gut. (A and B) Colonization kinetics (left), percentage string‐test negative (HMV‐) and rate of *Kp*VP loss (right) in colonies from stools collected from ampicillin‐treated mice colonized with P*rmpA* ^−T^ in (A) ampicillin or (B) PEG‐treated mice over 14 days. (C, D) Colonization kinetics (left), percentage string‐test negative (HMV‐) and rate of *Kp*VP loss (right) in colonies from stools collected from ampicillin‐treated mice colonized with *rmpA* ^+G^ coding sequence mutant in (C) ampicillin or (D) PEG‐treated mice over 14 days. Where no colony was enumerated at the limit of detection, data were plotted as 1 on the log axis. (E, F) Competition indices between SGH10‐GmR and SGH10 wild‐type, *Kp*VP‐null SGH10, *rmpA* ^+G^ CDS mutant, and P*rmpA* ^−T^ mutant co‐colonized in vivo in (E) ampicillin‐ or (F) PEG‐treated mice. (G) Colonization kinetics of SGH10 and the corresponding percentage loss of hypermucoviscosity detected in colonies from stools of mice pre‐treated with PEG, followed by 3 days of ampicillin treatment at 7 dpi. The limit of detection for HMV loss by the string test and *Kp*VP loss is 5%. Data in (E) and (F) were log_10_‐transformed and analyzed by two‐way mixed‐effects model, with Tukey's multiple‐comparisons test. Each point denotes an individual mouse. Data are shown as geometric mean ± geometric SD for dot plots. Means are represented in bar charts. Timepoints on the *x*‐axis of (A–D and G) are not plotted to scale.

To determine the functional consequences of the identified regulatory mutations, we used a clone containing the promoter region contraction (11 T → 10 T; P*rmpA* ^−T^) isolated from mouse stool and also engineered an isogenic SGH10 mutant with a similar + 1 frameshift mutation in the coding region of *rmpA* (*rmpA* ^+G^). Both types of mutant exhibited loss of mucoidy comparable to that of the *rmpA* deletion mutant (Δ*rmpA)* and had no apparent growth defect in vitro (Figure S3A,B). In both the ampicillin and PEG‐treated models, both mutants established robust colonization. Similar to wild‐type SGH10, both mutants maintained gut loads over 14 days in the ampicillin model and showed gradual decolonization in the osmotic perturbation model (Figure 4A–D). We did not observe in vivo reversion of either mutant to the wild‐type phenotype in the ampicillin or PEG‐treated gut over 14 days by string test. However, mutants colonized in the ampicillin‐treated mice showed some tendency to lose the *Kp*VP plasmid, unlike in PEG‐treated mice, where the plasmid appeared to be stably retained over time (Figure 4A–D).

Given that the hypermucoviscous capsule is known to be essential for immune evasion during inflammation, we postulated that an inflamed gut environment could drive reversion. We induced moderate colitis with 2.5% dextran sulfate sodium in mice colonized with the P*rmpA* ^−T^ and *rmpA* ^+G^ mutants of SGH10 to look for revertants in the gut. Among mice colonized with P*rmpA* ^−T^, 1 out of 5 harbored revertants (10 T → 11 T) at an allele frequency of 0.077 (Figure S3C), and among *rmpA* ^+G^ mutant‐colonized mice, 3 out of 5 mice carried mutants bearing a single‐nucleotide deletion within the poly‐G tract, restoring full‐length *rmpA* translation, with allele frequencies ranging from 0.04 to 0.096 (Figure S3D,E). This indicates that reversion at this locus remains rare even under inflammatory pressure in the gut.

We next quantified the fitness benefit of these mutations via competitive co‐infection in both models. In the ampicillin treatment model, control experiments confirmed neutral competition between wild‐type SGH10 and a gentamicin‐resistant wild‐type isogenic strain SGH10::Gm^R^ (Competitive Index (CI) of ~1 at Days 1 and 3) (Figure 4E). Similarly, in the osmotic diarrheal model, neutral competition was established between wild‐type SGH10 and isogenic SGH10 with chromosomally‐encoded sfGFP (SGH10::sfGFP) (Figure 4F). Strikingly, it was only in the ampicillin‐treatment model that the *rmpA* ^+G^ and P*rmpA* ^−T^ mutants (which retain the *Kp*VP plasmid) demonstrate competitive advantages identical to the plasmid‐cured strain (CI of ~10 at Day 1; CI of ~100 at Day 3) (Figure 4E). In the osmotic diarrheal model, the competitive advantage of P*rmpA* ^−T^ was maintained at ~10 up till Day 3, similar to the *rmpA* null mutant, whereas the *rmpA* ^+G^ mutant dropped to below 1 on Day 3 (Figure 4F). This demonstrates that the consequent loss of the HMV phenotype from both types of mutation is the primary driver of increased fitness in the ampicillin‐treated gut.

To explore the specific role of antibiotic pressure in selecting these variants, we subjected PEG‐treated mice to a secondary perturbation with ampicillin starting from 7 days post‐infection, predictably triggering resurgence of SGH10 in the gut (Figure 4G). The ampicillin‐induced bloom in PEG‐treated mice favored the emergence of HMV loss mutants and significantly extended their persistence through Day 14. However, the frequency of these variants remained lower than in the primary ampicillin model. A more rapid decrease in total bacterial loads was seen in the secondary ampicillin bloom compared to a primary ampicillin‐mediated colonization over 2 weeks. The fact that secondary ampicillin treatment was only administered for 3 days, and that the SGH10 population in the gut was already constrained by the initial PEG treatment, could certainly result in a different frequency of variants as compared to a primary ampicillin pre‐treatment model. PEG treatment could have led to a population bottleneck, resulting in fewer HMV‐loss variants emerging upon secondary ampicillin exposure.

### Distinct microbiome community structures created by antibiotic dysbiosis versus osmotic perturbation

To understand the ecological basis of the divergent colonization outcomes, we performed longitudinal long‐read metagenomics profiling to compare the microbiome community structures in mice treated with ampicillin or PEG for 5 days, followed by SGH10 infection. Stool samples were collected at three timepoints: Day 0 (post‐treatment, pre‐infection), Day 5 post‐infection, and Day 35 post‐infection. A naïve control group consisted of mice from the same batch, housed under identical conditions as the treatment group but receiving no treatment or infection during the 5‐day period. Their stool samples were collected at the same Day 0 timepoint as the treatment group.

**Figure 5:**
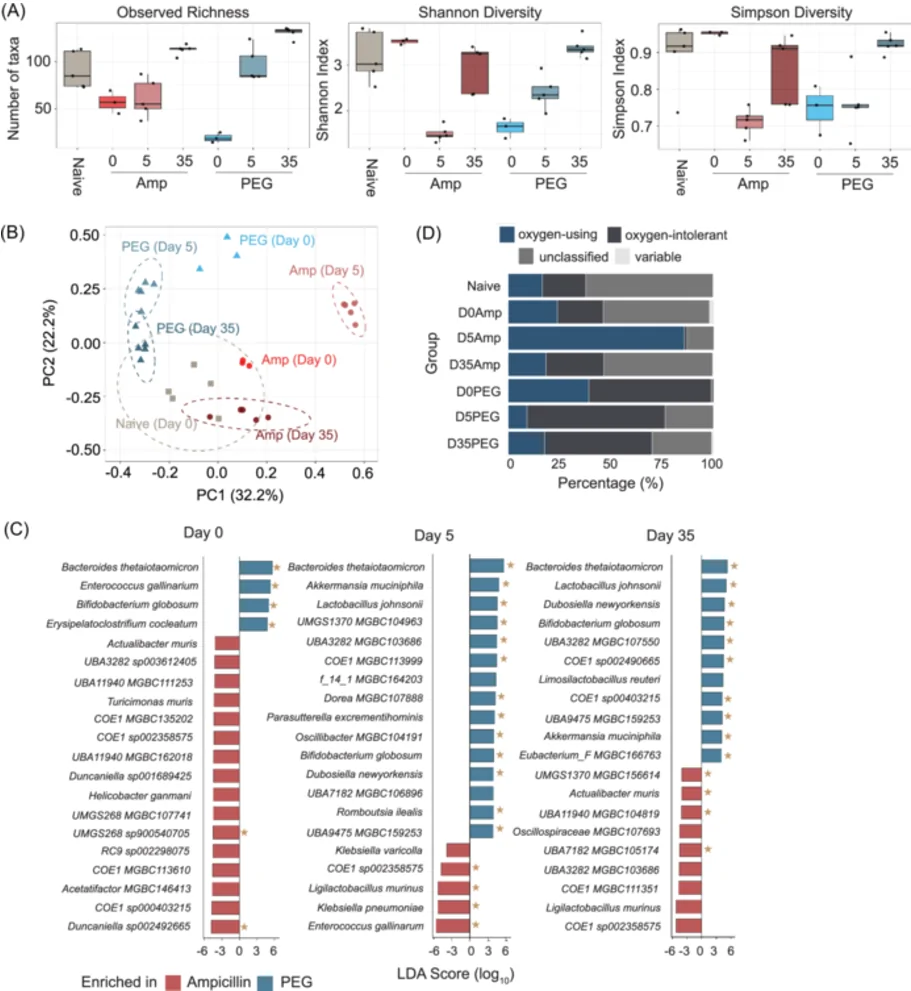
Distinct microbiome structures under ampicillin and PEG treatment during SGH10 colonization. (A) Alpha diversity metrics show observed richness, Shannon diversity, and Simpson diversity for each group. Each dot in the box plot represents the taxonomic composition of an individual mouse; the box center denotes the median, box limits represent the interquartile range (IQR), and whiskers extend to 1.5 × IQR. (B) Beta diversity visualized by PCoA, illustrating between‐group differences in Community composition. Dashed ellipses indicate 95% confidence intervals representing the dispersion of samples within each group. Each point denotes an individual mouse. (C) Linear discriminant analysis Effect Size (LEfSe) identifies differentially abundant bacterial species between ampicillin and PEG pre‐exposure groups at specific time points (Days 0, 5, and 35), with LDA scores indicating effect sizes. The star symbol indicates significance with the ANCOM‐BC2 test. (D) Oxygen‐tolerance analysis of the microbiome composition from each group based on BacDive classification. Genera were assigned to a group if ≥60% of entries supported the classification. Otherwise, they were labeled as variable. Those not identified in the MGBC database could not be taxonomically characterized and were labeled as unclassified. *Klebsiella* accounted for 22% of oxygen‐using species in the D5Amp group.

We observed an overall decrease in alpha diversity following treatment (Figure 5A). Compared to the naïve group, ampicillin treatment reduced species richness at Day 0 and Day 5, followed by recovery to higher richness by Day 35. Despite a marked reduction in total biomass (Figure 1C), ampicillin‐treated samples showed relatively high Shannon and Simpson diversity at Day 0 compared to naïve. By Day 5 post‐infection, both Shannon and Simpson diversity dropped sharply relative to naïve. This decline could be due to the expansion of *K. pneumoniae*, together with *Enterococcus gallinarum* and *Ligilactobacillus murinus* (Figure S4), which collectively accounted for ~80% of the community and established strong dominance. By Day 35, Shannon and Simpson indices increased to levels approaching those of the naïve group. For PEG treatment, richness was markedly reduced at Day 0 compared to naïve but recovered rapidly by Day 5 and further increased by Day 35 (Figure 5A). Both Shannon and Simpson diversity were substantially lower than naïve at Day 0, reflecting a community skewed toward a few dominant taxa, including *Bacteroides thetaiotaomicron*, *E. gallinarum*, and *Bifidobacterium globosum* (Figure S4). By Day 5, Shannon diversity increased but Simpson diversity remained largely unchanged, likely due to continued dominance by *B. thetaiotaomicron* (Figure S4). By Day 35, both Shannon and Simpson indices approached naïve levels, with *B. thetaiotaomicron* still representing a major species. However, while the Kruskal‐Wallis test showed significant overall differences in alpha diversity across groups for observed richness (*p* = 0.0003), Shannon (*p* = 0.0005), and Simpson diversity (*p* = 0.0025), post hoc pairwise comparisons using Wilcoxon tests with Benjamini–Hochberg correction did not reach statistical significance (Table S2), likely due to limited sample size and reduced statistical power. Thus, these changes in alpha diversity are best viewed as trends.

We then compared the taxonomic composition to assess differences between groups. There appeared to be differences in community composition between the two treatments, as supported by separate clustering in Bray‐Curtis dissimilarity (*R* ^2^ = 0.80, *p* = 0.001) (Figure 5B), although interpretation of the PERMANOVA analysis is limited by significant differences in multivariate dispersion (Table S3). The naïve baseline communities were dominated by Firmicutes and Bacteroidota (Figure S4A). Ampicillin treatment increased Firmicutes and promoted a Proteobacterial bloom, whereas PEG treatment favored Bacteroidota dominance. At the species level (Figure S4B), the naïve microbiome seemed dominated by Lactobacillaceae‐ and Lachnospiraceae‐affiliated taxa. Ampicillin treatment caused *L. murinus* to become dominant with blooms of *E. gallinarum* and *K. pneumoniae*, which were found to be significantly enriched compared to the PEG group at Day 5 (Figure 5C), resulting in a community enriched in oxygen‐using taxa (Figure 5D). By Day 35, the microbiome of the ampicillin group recovered to a more diverse state. In contrast, PEG treatment was associated with an expansion of the oxygen‐intolerant group (Figure 5D) such as saccharolytic and mucin‐associated species like *B. thetaiotaomicron*, *B. globosum*, and *Akkermansia muciniphila* (Figure S4B) that persisted up till Day 35. Notably, *B. thetaiotaomicron* was enriched with the highest relative abundance across all timepoints in the PEG group (Figure 5C).

Overall, our findings suggest that ampicillin treatment promotes a dysbiosis dominated by oxygen‐using taxa, including *Enterococcus, Klebsiella*, and *Firmicutes*, while PEG treatment favors a saccharolytic community enriched in anaerobes such as *Bacteroides, Bifidobacterium, Akkermansia*, and *Lactobacillus* species from pre‐treatment through post‐challenge recovery.

### Distinct microbiome‐exerted selection pressures are associated with divergent colonization and adaptation outcomes

We hypothesized that these distinct community architectures could impose divergent metabolic and competitive constraints on the invading pathogen. To empirically test whether these distinct macroenvironments directly modulate hv*Kp* fitness, we performed ex vivo growth assays using cecal contents. As cecal content immediately after ampicillin and PEG treatment harbors significantly fewer commensals and more water content respectively, we harvested caeca from mice that were allowed to recover from treatment for 2 days. Doing so allowed the ampicillin‐depleted microbiome to recover its biomass and PEG‐treated caeca to regain osmotic balance (Figure S5A–C). Metagenomic analysis of these cecal contents affirmed that despite 2 days of recovery, the species enriched in either group closely reflected those of Day 5 post‐infection (Figure 5C, Figure S5D,E).

**Figure 6:**
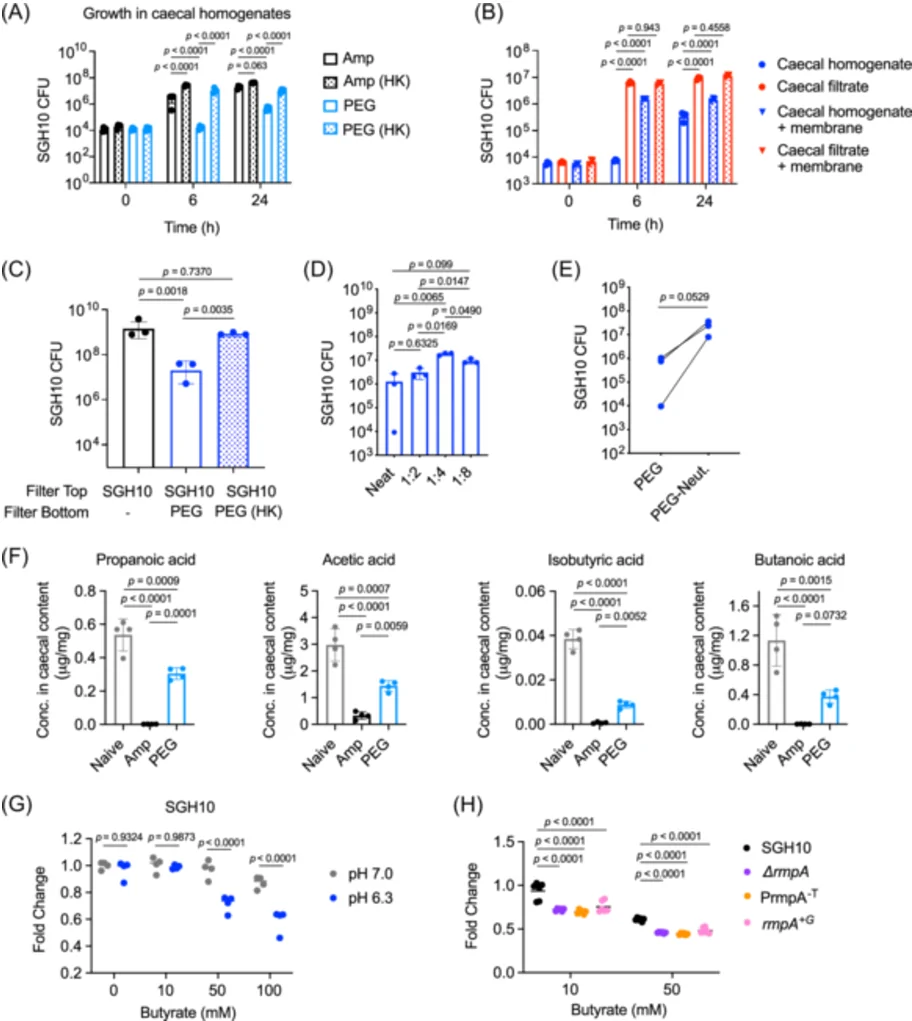
Microbiota‐mediated spatial proximity and metabolic selective pressure affect differential *K. pneumoniae* colonization and *rmpA* evolutionary trajectories. (A) SGH10 cultured in cecal homogenates generated from ampicillin‐ or PEG‐treated mice after 2 days of recovery with heat‐killed (HK) control and 100 mM MES supplementation under anaerobic conditions (*n* = 3). (B) SGH10 growth when cultured in cecal homogenates or bacteria‐free cecal filtrates generated from PEG‐treated mice after 2 days of recovery, with or without separation with 0.4 µm membrane under anaerobic conditions. Cecal homogenates from two mice were pooled for each replicate. (C) Cecal homogenates from PEG‐treated mice after 2 days of recovery cultured with SGH10 on BRM agar separated by a cellulose nitrate filter under anaerobic conditions. (D) SGH10 cultured in increasingly dilute spent cecal filtrates. (E) SGH10 cultured in overnight spent cecal filtrates versus neutralized filtrates. (F) SCFA quantification in cecal homogenates collected from naïve, ampicillin‐ and PEG‐treated mice after 2 days of recovery. (G) SGH10 cultured under anaerobic conditions in BRM media, with supplementation of 10 to 100 mM sodium butyrate for 6 h at pH 7.0 (control) or pH 6.3. Values were normalized to untreated conditions at pH 7.0 (*n* = 3). (H) SGH10 and its isogenic *rmpA* mutants were cultured under anaerobic conditions in BRM media, with supplementation of 10 or 50 mM sodium butyrate for 24 h at pH 5.75. Values were normalized to untreated conditions (*n* = 2 separate experiments were conducted in triplicate). Each point in (C–F) denotes an individual mouse. Data in (A) and (B) are shown as geometric mean ± geometric SD, and CFU were log10‐transformed and analyzed by two‐way ANOVA. Data in (C) and (D) are shown as geometric mean ± geometric SD, and CFU were log_10_‐transformed and analyzed by one‐way ANOVA. CFU data in (E) were log10‐transformed and analyzed by a paired *t*‐test. Data in (F) are shown as mean ± SD and analyzed by one‐way ANOVA. Data in (G) and (H) are shown as mean ± SD and analyzed by two‐way ANOVA.

Consistent with a permissive niche, cecal homogenates from ampicillin‐treated mice supported robust SGH10 proliferation at 6 h and 24 h post‐incubation (Figure 6A). In contrast, cecal homogenates from PEG‐treated mice exerted significant growth inhibition at 6 h with some growth recovery at 24 h that was still lower than that seen with ampicillin‐treated cecal homogenates. Heat inactivation of PEG‐treated cecal homogenates greatly diminished this inhibitory effect, and PEG‐treated cecal filtrates that contained no bacteria similarly lost the ability to inhibit SGH10, indicating that inhibition requires a live bacterial community. To determine whether this inhibition is contact‐dependent, we incubated PEG cecal homogenates with SGH10 while separating the two by a 0.4 μm filter in a transwell system; inhibition was abolished, suggesting that direct contact or close proximity between the live community and SGH10 is necessary for inhibitory activity (Figure 6B). Furthermore, while co‐culturing on solid medium maintained the inhibitory phenotype, no zone of inhibition was observed when spotting the homogenate onto a SGH10 lawn (Figure S5F,G).

We postulated that in the transwell setup (Figure 6B), SGH10 and the PEG cecal communities were still some distance apart and inhibitory factors could have been diluted or that the inhibitory metabolites were labile and needed to be produced continuously at short distance at high enough concentrations to be effective. When we instead co‐cultured cecal homogenates on solid agar, separated from SGH10 only by a filter, we once again observed inhibition of SGH10, supporting that direct contact was not necessary (Figure 6C). To amplify the effects of any inhibitory metabolites released by the PEG cecal community, we cultured the cecal homogenates anaerobically for 24 h, collected the spent culture filtrates, and cultured SGH10 in progressively PBS‐diluted filtrates to assess if inhibition would be lifted. SGH10 was able to grow in filtrates diluted up to eightfold, indicating that inhibition was not due to nutrient limitation (Figure 6D). In fact, dilution of spent filtrates permitted more growth of SGH10, suggesting the presence of inhibitory factors produced by the PEG cecal community (Figure 6D). We observed that SGH10 cultured with PEG cecal homogenates containing live communities resulted in a slightly acidified medium (~pH 6.3) than when cultured in cecal filtrate alone (~pH 6.6) despite buffering by MES (Figure S5H). Without buffering, the culture acidified to approximately pH 5.5, within the effective inhibitory range of short‐chain fatty acids (SCFAs); when the pH of the neutralized spent culture was raised to 7, inhibition of SGH10 was abrogated (Figure 6E). In the absence of SCFAs, a pH of 5.5 in LB or Bioreactor medium (BRM) alone did not inhibit SGH10 growth (Figure S5I). Together, these findings point to SCFAs as possible actors for inhibition of SGH10.

Indeed, PEG cecal content contained significantly more SCFAs than ampicillin‐treated cecal contents, although naïve caeca contained the highest amounts (Figure 6F, Figure S5J). Culturing SGH10 in butyrate‐supplemented medium demonstrated a sizeable inhibition even at pH 6.3 (Figure 6G), suggesting that SCFAs in PEG‐treated caeca could be responsible for suppressing SGH10 and promoting decolonization upon repopulation with SCFA‐producing commensals. Furthermore, *rmpA* mutants appeared more susceptible to SCFA‐mediated inhibition than wild‐type SGH10 (Figure 6H), which could drive selection against the establishment of these mutants in the PEG cecal environment.

## DISCUSSION

Gut microbiome dysbiosis is often thought of as a binary state that uniformly promotes pathogen expansion and colonization [20, 21]. However, our findings demonstrate that distinct perturbation modalities generate fundamentally divergent macroenvironments, each imposing unique selective pressures that dictate not only the persistence of hv*Kp* but also its evolutionary trajectory.

Antibiotic treatments have been shown to predispose to pathobiont colonization for various durations of time. Studies have shown that some individuals experience slow microbiome recovery even 1 year after antibiotic treatment [22, 23, 24]. A recent study reported that for certain antibiotics, previous exposures of even 4–8 years prior could cause a lasting alteration of microbiome architectures [25]. In our model, transient antibiotic exposure with ampicillin resulted in a state of high density and prolonged hv*Kp* colonization, long after the antibiotics were removed. This state is characterized by the collapse of obligate anaerobes and the expansion of facultative opportunists such as *Enterococcus* and *Ligilactobacillus*. The transient inflammatory signals we detected, and the expansion of oxygen‐using species, point to a redox shift to a more oxidative environment that is conducive for facultative anaerobes such as Enterobacteriaceae species, as described by others [26]. Conversely, osmotic perturbation by laxative administration, frequently used for constipation or colon‐cleansing during colonoscopy procedures, created a state of “resilient dysbiosis” functionally distinct from antibiotic‐induced dysbiosis. Despite initial physical washout, we postulate that the rapid recovery of a saccharolytic, mucin‐specialist community (dominated by *B. thetaiotaomicron*, *Bifidobacterium*, and *Akkermansia*) mediated the swift decolonization of hv*Kp*. This aligns with reports identifying *B. thetaiotaomicron* as a keystone species that enforces colonization resistance through cross‐feeding and establishment of secondary and tertiary colonizers [27] mediating intense nutrient and interference competition that inhibit pathogen growth. Consistent with the differential microbiome composition between the two modalities, we found much higher production of SCFAs in the PEG‐treated cecal contents, with negligible production in the antibiotic‐treated samples. The heat sensitivity and requirement for close proximity for the PEG‐cecal contents to work likely indicate that SCFAs needed to be at high enough concentrations achievable only within an acidified microenvironment in close proximity to the pathogen to actively inhibit hv*Kp* growth.

What is previously unappreciated is our discovery that these distinct microbiome ecological states drive divergent evolutionary outcomes via a virulence‐persistence trade‐off. The polysaccharide capsule is a metabolically expensive virulence factor, consuming significant ATP and carbon precursors [28, 29]. In the nutrient‐rich, competitor‐depleted environment of the ampicillin‐treated gut, the benefits of capsule protection might be minimized, while the metabolic cost becomes a liability. Consequently, we observed rapid selection for HMV‐loss variants in the population where loss of the entire *Kp*VP plasmid contributed only 10%. The major mechanism of HMV loss is attributed specifically to variants in the *rmpA* locus. Indeed, the *rmpA* promoter and coding sequence variations found were detected at a much higher frequency than other capsule loci mutations (see Table S1). The recurrence of expansions or contractions in the poly‐T tract of the *rmpA* promoter and poly‐G tract of the *rmpA* coding sequence suggests these regions function as phenotypic switches, employing slippage‐mediated capsule phase variation as a possible bet‐hedging strategy. The higher prevalence of *rmpA* variants rather than whole plasmid loss is likely advantageous because loss of the entire *Kp*VP would abrogate other plasmid‐encoded virulence or metabolic functions, thereby constraining the capacity to flexibly adapt to changes in the environment. Moreover, this adaptive loss of HMV likely enhances fitness by redirecting metabolic flux toward growth, enabling the pathogen to maximize its carrying capacity in the permissive niche. Indeed, capsule‐loss mutants have been frequently observed during *in vitro* experiments in nutrient‐rich media [28], likely due to metabolic trade‐offs in order to maximize growth when nutrients are abundant. However, we do not commonly see capsule loss in hv*Kp* in the gut, in agreement with another report [15]. Additionally, this potentially reversible mutational strategy preserves the ability of the bacteria to transit out of the gut into other niches where preserving HMV capsule production is a matter of life and death, as *rmpA* is a key determinant of virulence during systemic infection [30, 31].

In stark contrast, the competitive environment of the PEG‐treated gut imposes strong purifying selection where the metabolic stress exerted by the recovering microbiome occurs at two levels. Firstly, the “resilient microbiome” produces SCFAs that constrain the pathobiont's growth, evident by a lower colonization load that diminishes over time. Secondly, the stronger inhibitory effect of SCFAs on HMV‐loss mutants relative to wild‐type bacteria reduces the opportunity for their establishment and for the mutation to sweep the population to achieve fixation. This conclusion is reinforced by our secondary ampicillin perturbation, where in a formerly PEG‐exposed gut, the frequency of these variants remained lower than in the primary infection. This is likely due to a population bottleneck. Unlike the primary ampicillin model, where the inoculum enters a sparsely occupied niche from a population grown in vitro, the secondary bloom in the PEG–ampicillin model likely arises from a small in vivo founder population. Under secondary ampicillin selection, this small population could be dominated by the wild‐type strain during early expansion, delaying the fixation of adaptive variants. Furthermore, when the *rmpA* mutants were competed against the wild‐type in ampicillin‐treated versus PEG‐treated gut, their competitive advantage was significantly lower in PEG than in the ampicillin model. This likely underscores why significantly more HMV loss variants were observed in the ampicillin‐reshaped gut as the adaptive mutation achieves fixation in some animals, whereas in the PEG‐treated animals, these variants did not reach high enough frequency to sweep the population. Our findings that the *rmpA* mutants were more susceptible to the inhibitory action of butyrate compared to wild‐type bacteria suggest that capsule thickness and mucoidy could constitute a steric barrier that slows the entry of SCFAs into bacterial cells.

Importantly, the *rmpA* poly‐T promoter variants have been described in clinical isolates from patients in China, demonstrating the relevance of our work to real‐life infection scenarios. One study found the P*rmpA* ^−T^ mutation in a ST23‐KL1 patient isolate [32], while another group also reported this mutation in many ST11‐KL64 isolates from sputum, feces, and blood [33]. Repeated sampling of ST11‐KL64 isolates bearing the virulence plasmid from the sputum of a patient over a period of 29 days in the intensive care unit revealed isolates with the P*rmpA* ^−T^ mutation in the last two sampling time‐points. The time at which the patient exhibited recovery correlated to when the infecting isolate had transitioned to an HMV‐loss phenotype [34]. These examples of HMV‐loss variants in different infection niches under constant antibiotic selection pressure experienced by the hospitalized patients could demonstrate a more general principle of the trade‐off between persistence versus virulence under antibiotic selection. Additionally, the coding sequence variant was also observed recently by Stawarska et al. (bioRxiv) [35]. They demonstrated that such mutants could undergo phase variation, with a subpopulation reverting back to the wild‐type phenotype in vitro. We did not observe reversion in the promoter or the coding region of the *rmpA* mutants in the ampicillin‐ or PEG‐treated gut context. However, given that our sampling strategy had a significant limit of detection (5%) for HMV^+^ colonies by string test, revertant bacteria that could have arisen in the gut may have flown under the radar. Using a colitis model to induce gut inflammation, we observed low frequencies of revertant mutants in the stools of colonized mice by variant calling. Together, these findings suggest that the phase‐variable nature of the hypermucoviscous capsule could be part of hv*Kp*'s strategy for adaptation and survival in different host niches. Future work could explore this variation in other contexts, such as during gut translocation to systemic circulation, and defining the dynamics and physiological triggers modulating phase variation directionality, which could be exploited for hv*Kp* infection control and treatment.

While our study offers a compelling look at how different modes of gut perturbation influence the evolutionary fate and colonization of *K. pneumoniae*, several limitations of the study exist. First, it is conceivable that different classes of antibiotics with diverse mechanisms of action and bacterial spectra could have differential effects from what we observed with ampicillin treatment [25]. Studies on healthy donors and patients undergoing bowel preparations for colonoscopy with various PEG regimens demonstrated that some patients showed no dysbiosis while others had temporary or prolonged reductions in gut microbial diversity [36, 37]. Hence, our results observed with PEG may not generalize to all osmotic laxative regimens, nor to other classes of laxatives such as stimulant laxatives, stool softeners, or bulk‐forming agents. Furthermore, we had only used one dosage and a fixed 5‐day duration of antibiotic pre‐treatment to simulate a short intensive treatment course. It is possible that modulating the duration and dose would change the severity of dysbiosis, thereby affecting the recovery speed of a resilient microbiome. Our relatively small sample size for microbiome analysis also limits the statistical power of the post‐hoc pairwise comparisons of alpha diversity, as well as definitive separation of microbial communities by beta diversity. However, this limitation does not alter the primary conclusions that distinct perturbations led to different ecological outcomes supported by community and metabolite changes.

## CONCLUSION

Overall, our findings reshape our understanding of gut microbiome and pathogen interactions under dynamic physiological changes. The gut is not merely a passive reservoir but a dynamic arena where the type of disturbance shapes the evolutionary landscape. Transient exposure of common beta‐lactam antibiotics such as ampicillin is associated with long‐term persistence of the hv*Kp* pathobiont in the gut, while osmotic laxative PEG3350 causes only a transient decrease in richness and preserves the “resilient” microbiome architecture, resulting in colonization resistance against pathobionts. Antibiotic exposure favors the emergence of “commensal‐like” phenotypes, trading acute pathogenicity for chronic persistence. Therefore, understanding the unintended consequences of even transient antibiotic exposure is essential for developing strategies to mitigate these effects. Collectively, these data advocate for antibiotic stewardship that includes greater consideration of the long‐term evolutionary consequences of microbiome perturbation and pathogen evolution.

## METHODS

### Bacterial strains and culture

*K. pneumoniae* strain SGH10 (Accession no.: CP025080), NUH61 (Accession no.: JARYXD000000000), and SGH07 (Accession no.: CP124521.1) and their isogenic mutants were grown on Lysogeny Broth (LB) agar and in LB broth. For oral inoculation into mice, bacterial counts in overnight culture were determined by OD600 measurement and strains were diluted in PBS accordingly. For anaerobic experiments, SGH10 was cultured in Bioreactor media (BRM) or on BRM agar (see Supplementary Methods), which mimics the nutrient‐sparse conditions of the distal colon [38]. BRM was pre‐incubated overnight inside an anaerobic chamber to remove oxygen.

### Construction of isogenic mutants in *K. pneumoniae* SGH10

Gene deletion in *K. pneumoniae* strains was performed as previously described [39]. Briefly, Phanta Flash DNA polymerase (P520‐03, Vazyme) amplified upstream and downstream regions of the target gene were assembled with pR6KmobsacB using NEBuilder® HiFi DNA Assembly Master Mix (E2621, New England Biolabs) and then introduced into the *K. pneumoniae* strain through conjugation using *Escherichia coli* S17‐1 λpir as a donor. Selection was performed with 100 μg/mL kanamycin (K1377, Sigma‐Aldrich) and 40 μg/mL fosfomycin (P5396, Sigma‐Aldrich). Negative selection was performed by overnight culturing in LB with 20% sucrose and then spreading onto LB agar plates. Viable colonies were screened by PCR, followed by nucleotide sequencing (1st Base, Singapore) to validate gene deletion. To engineer isogenic SGH10 mutants carrying the *rmpA* coding sequence insertion (10 G → 11 G; *rmpA* ^+G^), we created the *rmpA* mutation in pR6KmobsacB and introduced it into SGH10Δ*rmpA* by conjugation. Sucrose counterselection was performed, and colonies were screened for *rmpA* reversions by PCR, followed by nucleotide sequencing to validate gene mutations. SGH10::sfGFP was created by inserting the sfGFP gene into the *lacY* locus. The *lacY* upstream, *sfGFP* gene, and *lacY* downstream regions were assembled in pR6KmobsacB and then introduced into *K. pneumoniae* SGH10. Sucrose counterselection was performed, and colonies were screened for sfGFP insertion by PCR. SGH10::Gm^R^ was created by inserting a Gm^R^ gene in *glmS* locus using the Tn7 delivery vector pGRG36 [40].

### Antibiotic and PEG3350 perturbation gut colonization models

Female C57BL/6 mice (InVivos, Singapore), aged 7–8 weeks, were used in all experiments. All mice were maintained in a pathogen–free animal biosafety level 2 facility at the National University of Singapore, in compliance with National Advisory Committee for Laboratory Animal Research (NACLAR) guidelines and acclimatized for 3 days. For the antibiotic perturbation model, mice were pre‐administered 2.5 mg of ampicillin (A9518, Sigma‐Aldrich) daily by oral gavage for five consecutive days in 100 μL of PBS. For the osmotic perturbation model, mice were given sterile 15% polyethylene glycol (PEG) 3350 (MiraLAX®) in their drinking water *ad libitum* for five consecutive days, then replaced with non‐supplemented water. For mono‐infection experiments, overnight cultures of *K. pneumoniae* strains and their mutants were diluted to 1 × 10^6^ CFU and inoculated into mice via oral gavage in 100 μL of PBS.

In vivo bacterial competition was performed using distinct reference strains depending on the perturbation model. In the ampicillin model, 5 × 10^5^ CFU of SGH10‐GmR and 5 × 10^5^ CFU of the mutant of interest were prepared in 100 μL PBS and inoculated into mice via oral gavage. In the PEG model, competition was performed using an isogenic SGH10 with chromosomally encoded sfGFP (SGH10::sfGFP) as the reference competitor. Stool pellets were collected in PBS at each time point during colonization, weighed, homogenized, and vortexed before being serially diluted and plated on LB agar supplemented with 100 μg/mL carbenicillin to enumerate *K. pneumoniae* colonization loads, on LB agar supplemented with 100 μg/mL carbenicillin and 50 μM potassium tellurite (Sigma‐Aldrich, P0677) to enumerate *Kp*VP^+^ bacterial colonization loads, and on LB agar supplemented with 15 μg/mL gentamicin (G1264, Sigma‐Aldrich) to enumerate SGH10‐GmR colonization loads. SGH10::sfGFP colonies were identified and enumerated based on GFP fluorescence using an iBright™ Imaging System (Thermo Fisher Scientific).

### Colon tissue collection and imaging

A portion of the proximal colon was dissected and flash‐frozen in OCT freezing medium. Samples were cryo‐sectioned into 5 μm slices and mounted on charged slides. For H&E staining, flash‐frozen colon sections were dried for 1 h at room temperature before staining with haematoxylin (1.05174.0500, Merck) for 2 min and counterstaining with eosin (HT110116, Sigma‐Aldrich) for 10 s. Sections were dehydrated in 70%, 95%, and 100% ethanol, dipped in 4 changes of xylene, and mounted using Dibutylphthalate Polystyrene Xylene (DPX) Mountant (06522, Sigma‐Aldrich). For Alcian blue staining, slides were incubated in 3% acetic acid for 3 min, then stained with Alcian blue solution (Sigma‐Aldrich, B8438) for 30 min, washed in running tap water for 2 min, and mounted. Images were captured on an EVOS M7000 (AMF7000, Thermo Fisher Scientific). H&E and Alcian blue‐stained images were acquired using a 10× and 4× objective, respectively.

### Intestinal CFU loads enumeration

Ampicillin‐ or PEG‐treated mice were colonized with *K. pneumoniae* strain SGH10, and the gastrointestinal tract was harvested from the small intestine to the distal colon at 3, 10, and 35 dpi. Tissue was separated into intestinal segments: duodenum, jejunum, ileum, caecum, and colon, with digestive matter intact, and transferred into tubes containing 2 mL of PBS for homogenization with 2.8 mm beads (19‐678, OMNI International), twice at medium speed for 30 s to liberate bacterial cells. Tissue homogenates were then serially diluted and plated on LB plates supplemented with carbenicillin for CFU enumeration.

### Colonic lamina propria isolation and immune cell profiling

Colons were harvested from mice and placed in cold RPMI with 5% FBS. Fecal content was removed from the colon, and the tissue was cut open longitudinally and gently scraped to remove residual contents and loose mucus. The colon tissue was then cut into 5 mm pieces, rinsed with PBS, and incubated in dissociation buffer containing HBSS, 2 mM EDTA, and 10 mM HEPES, shaking at 200 rpm for 15 min at 37°C. Tubes were shaken vigorously, and the contents were decanted onto a 70 μm nylon mesh and rinsed with HBSS. Tissue pieces were incubated in dissociation buffer again for 15 min, transferred into 10 mL pre‐warmed digestion buffer containing RPMI, 1X sodium pyruvate, 1X MEM‐NEAA, 10% FBS, 2 mg/mL collagenase IV, and 0.05 mg/mL DNAse I. Tissue pieces were incubated with shaking at 200 rpm at 37°C for 45 min, with vigorous manual shaking at 8 min intervals. Digested tissue was then strained through a 70 μm filter and washed with HBSS containing 2 mM EDTA before centrifuging at 500 g for 5 min at 4°C to pellet lamina propria cells. Cells were aliquoted into 96‐well plates for flow cytometry staining. Cells were washed with PBS prior to staining with the Zombie NIR™ Fixable Viability Kit (423105, Biolegend) for 20 min, then washed with FACS buffer (1X PBS, 2% FBS, 2 mM EDTA), then blocked with anti‐CD16/32 at 4°C for 15 min. Next, cells were stained with a biotin‐labeled antibody cocktail at 4°C for 25 min, followed by washing and staining with an extracellular antibody cocktail at 4°C for 25 min. After a final wash, cells were fixed with 2% PFA for 15 min at RT, washed, and re‐suspended in FACS buffer prior to acquisition on a BD LSRFortessaTM X‐20. Analysis was performed in FlowJo 10.8.2. The gating strategy and antibodies used for staining can be found in the Supplementary Information.

### Cecal content DNA extraction

Cecal contents from mice were harvested and a portion of the cecal content was dried for 48 h at 40°C, and the water weight percentage of each sample was calculated from the wet and dry weights. DNA was extracted from the remaining cecal content using the QIAamp PowerFecal Pro Kit (51804, Qiagen). DNA concentration per dry weight was calculated by accounting for the previously recorded water weight percentage of each sample.

### Hypermucoviscosity assay

Hypermucoviscosity was determined by performing a string test on bacterial colonies grown on solid agar, with a positive result defined as the formation of a viscous capsular string >5 mm in length. All colonies exhibited similar morphology and were therefore selected at random for the string test (Figure S2A). String test negative colonies were randomly selected for further confirmation using a low‐speed sedimentation assay (5 min, 2000 × *g*) from an overnight LB culture, yielding a tight bacterial pellet. String test negative colonies were spotted onto LB supplemented with 50 μM potassium tellurite to determine whether they retained the *Kp*VP plasmid.

### Variant calling

*K. pneumoniae* strain SGH10 was gavaged into mice pre‐treated with ampicillin or PEG for 5 days, and stool samples were collected on Days 0 (pre‐gavage), 5, 7, and 14. Stools were homogenized, plated on LB agar with carbenicillin to select for SGH10 lawns, and scraped for DNA extraction. Samples from the PEG group at Days 7 and 14 were excluded due to very low bacterial counts. Extracted DNA was subjected to paired‐end Illumina sequencing, yielding average chromosomal coverage of 512.5 ± 53.5 and *Kp*VP1 coverage of 242.2 ± 60.8 (mean ± SD). Reads were aligned to reference genomes (NZ_CP025080.1 for the SGH10 chromosome and NZ_CP025081.1 for *Kp*VP1), and variants including single‐nucleotide variants and indels were identified, quality‐filtered, and annotated using the LoFreq (v2.1.5) pipeline [41], with allele frequencies computed from the filtered calls. Variants present in the original in vitro bacterial inoculum (input control) were removed from all other samples to ensure that only sample‐acquired variants remained.

### ONT library preparation and sequencing

Mice were colonized with SGH10 after pre‐treatment with 5 days of ampicillin or PEG as previously stated (*n* = 5 animals). Stools were collected from mice pre‐treatment, at the end of 5 days of either ampicillin or PEG treatment, and at 5 and 35 days post SGH10 colonization after both treatments, for gDNA extraction and metagenomic sequencing. Five random samples among naive stools from the same group of mice were selected for sequencing. DNA extraction from mouse stool samples was performed using QIAamp PowerFecal Pro Kit (Qiagen). DNA concentration was quantified using the Qubit™ 1X dsDNA High Sensitivity (HS) (Q33231, Invitrogen) or Broad Range (BR) Assay Kits (Q33266, Invitrogen) on a Qubit 4 Fluorometer (Q33238, Thermo Fisher Scientific). Due to low yields of bacterial DNA after ampicillin and PEG treatment, some samples were pooled for sequencing, and *n* = 3 samples remain for Day 0 Post‐Amp and Post‐PEG groups. Sequencing libraries were prepared using the Native Barcoding Kit 24 V14 or Native Barcoding Kit 96 V14 (SQK‐NBD114.14 or SQK‐NBD114.96, Oxford Nanopore Technologies), with minor modifications to the manufacturer's protocol. Briefly, DNA was used directly for DNA repair and end‐prep to preserve fragment integrity, and the native barcode ligation step was extended from 20 min to 2 h. Samples were pooled to achieve equal DNA input amounts (ng) prior to adapter ligation. The final library was sequenced on a MinION Mk1C platform using R10.4.1 flow cells (FLO‐MIN114, Oxford Nanopore Technologies) for up to 72 h. Raw signal data were basecalled using the latest available version at the time of sequencing (Guppy v6.4.2 or Dorado v1.3.1) with the super‐high accuracy (SUP) model. Basecalled reads were subsequently demultiplexed, and adapter and barcode sequences were trimmed prior to downstream analysis.

### Microbiome taxonomic composition analysis

Metagenomic reads were taxonomically classified using Kraken2 (v2.1.2) [42] with the Mouse Gastrointestinal Bacterial Catalogue (MGBC) database [43] (parameters: ‐‐minimum‐hit‐groups 3 ‐‐report‐minimizer‐data ‐‐confidence 0.1). Species‐level abundance estimates were subsequently generated using Bracken (v3.0.1) [44] (parameters: ‐r 150 ‐l S ‐t 10). Species with a mean relative abundance of at least 0.1% in any group were retained, while lower‐abundance taxa were grouped into “Other”. Phylum‐level assignments were derived from the corresponding species‐level classifications. Alpha diversity (Observed, Shannon, and Simpson indices) was calculated in R (version 4.4.3) using the *vegan* package (v2.7.2) and compared by Kruskal–Wallis and pairwise Wilcoxon tests with Benjamini–Hochberg correction. Beta diversity was estimated using Bray–Curtis dissimilarity, visualized by principal coordinates analysis (PCoA), and tested by PERMANOVA (999 permutations, *a* = 0.05). All visualizations were generated in R using *ggplot2* (v4.0.0) and related packages. A fixed random seed of 7 was used to ensure reproducibility of permutation‐based analyses. Differential abundance analysis was performed using LEfSe (v1.1.2) [45] and further validated with ANCOM‐BC2 (v2.0.1) [46]. The results were parsed and visualized in R, generating bar plots of LDA scores showing significantly enriched taxa for each comparison and labeled with stars if supported by ANCOM‐BC2. Oxygen tolerance was annotated using the BacDive database (retrieved on 19 Jan 2026) [47]. Categories were consolidated into oxygen‐using (aerobes, facultative aerobes, facultative anaerobes, microaerophiles, obligate aerobes), oxygen‐tolerant (aerotolerant, microaerotolerant), and oxygen‐intolerant (obligate anaerobes). For each genus, the predominant classification was determined based on the proportion of entries supporting a given group. A genus was considered to belong to a group when this proportion reached ≥60%; genera without a predominant classification were considered variable. Genera absent from the MGBC database were designated as unclassified if their taxonomic characteristics could not be determined.

### SCFA inhibition assay

Sodium butyrate (10, 50, and 100 mM; 303410, Sigma‐Aldrich) was dissolved in BRM, and the supplemented media were pH‐adjusted using 1 M hydrochloric acid (HCl) or sodium hydroxide (NaOH). All media were sterilized by filtration through a 0.22 µm pore‐size membrane filter (SLGPR33RS, Millipore, Burlington, MA). Overnight bacterial cultures were subcultured into fresh BRM or BRM supplemented with sodium butyrate at the indicated concentrations and pH conditions. Overnight bacterial cultures were diluted to an initial OD_600_ of 0.01 (approximately 1.5 × 10^7^ CFU/mL). Cultures were incubated statically for 24 h at 37°C within an anaerobic chamber. Bacterial growth was quantified by measuring OD600 using a microplate reader.

### *Ex vivo* cecal static culture assay

Mice were treated with ampicillin or PEG for 5 days, followed by a 2‐day recovery period. Mice were euthanised by CO_2_ inhalation followed by cervical dislocation. The caecum was aseptically excised and transferred into an anaerobic chamber under an atmosphere of 5% H_2_, 5% CO_2_, and 90% N_2_. Cecal contents were collected and diluted four‐fold in pre‐reduced PBS. Samples were homogenized by vortexing and pipetting with wide‐bore tips to ensure uniformity. All buffers and reagents used in the assay were pre‐reduced in the anaerobic chamber for at least 24 h before use. For heat‐killed controls, cecal homogenates from post‐ampicillin and post‐PEG treatments were incubated at 75°C for 30 min. Cecal homogenates were dispensed into 96‐well plates containing 100 mM pre‐reduced 2‐(N‐Morpholino) ethanesulfonic acid (MES) buffer. Subsequently, *K. pneumoniae* SGH10 suspension was added to achieve a final inoculum of approximately 1 × 10^4^ CFU per well. Plates were incubated statically at 37°C under anaerobic conditions. At the appropriate timepoints, a 10 μL aliquot was collected from each well and plated on LB plates supplemented with 100 μg/mL carbenicillin for CFU enumeration. The 96‐well Transwell assay was adapted from Pickard et al. [48], using 0.4 μm pore size polycarbonate membrane inserts (CLS3381, Sigma‐Aldrich). In this setup, 75 μL of cecal filtrate containing *Kp* SGH10 was added to the upper insert, while 235 μL of either whole cecal homogenate or cecal filtrate, both supplemented with 100 mM MES buffer, was placed in the lower well. In parallel, direct contact controls involved inoculating *Kp* without a membrane barrier. For solid agar filter separation experiments, PEG cecal homogenates or heat‐killed controls were spotted onto a cellulose nitrate filter (11406‐47‐ACN, Sartorius, Göttingen, Germany) laid on BRM agar. A second filter was layered on top, and SGH10 (1 × 10^4^ CFU) was spotted onto the upper filter. Following incubation, both filters were removed and vortexed vigorously in 1 mL PBS for CFU enumeration. For experiments using spent cecal filtrate, caeca were harvested from mice treated with PEG for 5 days, followed by a recovery period of 2 days. The resulting cecal homogenates were centrifuged, and supernatants were filtered through a 0.2 μm membrane. Spent filtrates were either neutralized with 1 M NaOH or diluted with PBS and subsequently used to culture 1 × 10^4^ CFU SGH10 statically at 37°C anaerobically for 6 h, followed by bacterial enumeration.

### Cecal SCFA quantification

Mice were treated with ampicillin or PEG for 5 days, followed by a 2‐day recovery period. Cecal contents were then harvested and immediately flash‐frozen in liquid nitrogen. Samples were sent to 1st BASE for SCFA quantification by gas chromatography‐mass spectrometry (GC‐MS). Detailed methods are described in the Supplementary Methods.

### Statistical analysis

All statistical analyses were performed using GraphPad Prism (version 11.0.2). CFU and competitive index data were log_10_‐transformed before statistical analyses. Where no colonies were enumerated, data were plotted as 1 on the log axis. Data involving repeated measurements from the same animals at multiple time points were analyzed using a two‐way mixed‐effects model, with treatment group and time as fixed effects and individual mouse as the repeated‐measures subject. Sidak's or Tukey's multiple‐comparisons test was used for post hoc comparisons between treatment groups at individual time points. Experiments involving independent animals at each time point were analyzed using a conventional two‐way ANOVA with Sidak's multiple‐comparisons test. Static co‐culture experiments and in vitro SCFA inhibition assays were also analyzed by two‐way ANOVA. Comparisons of cecal content weight, biomass, bacterial counts, SCFA concentrations, and hypermucoviscosity between SGH10 and its isogenic mutants were performed using one‐way ANOVA for more than two groups, or unpaired *t*‐test for two groups.

## AUTHOR CONTRIBUTIONS

**Carey Lim**: Investigation; writing—original draft; writing—review and editing; methodology; validation; formal analysis; conceptualization. **Jialing Zheng**: Investigation; methodology; validation; formal analysis; writing—review and editing. **Guoxiang Cheam**: Investigation; writing—review and editing; methodology; validation; formal analysis. **Melvin Yong**: Data curation; investigation; writing—original draft; writing—review and editing; formal analysis; validation. **Hanrong Chen**: Formal analysis; validation; writing—review and editing. **Andrew Ting**: Investigation. **Yahua Chen**: Methodology; writing—review and editing. **Qingqing Wang**: Resources; project administration; writing—review and editing. **Niranjan Nagarajan**: Formal analysis; resources; writing—review and editing. **Yunn‐Hwen Gan**: Supervision; resources; project administration; formal analysis; validation; conceptualization; writing—original draft; writing—review and editing; funding acquisition.

## CONFLICT OF INTEREST STATEMENT

The authors declare no conflicts of interest.

## ETHICS STATEMENT

All experiments involving mice were performed in accordance with the recommendations in the Guide for the Care and Use of Laboratory Animals. The protocol was approved by the Institutional Animal Care and Use Committee (IACUC) of the National University of Singapore (Protocol No. R21‐1498).

## Supporting information


**Figure S1:** Ampicillin and laxative models induce a differential colonic immune response with SGH10 colonisation.
**Figure S2:** Loss of HMV phenotype in stool bacteria from ampicillin‐treated mice.
**Figure S3:** In vitro phenotype of SGH10 P*rmpA* ^−T^ and *rmpA* ^+G^ mutants and *in vivo* reversion in induced colitis model.
**Figure S4:** Distinct microbiome structures under ampicillin and PEG treatment during SGH10 colonisation.
**Figure S5:** Caecal homogenates from PEG‐treated mice after 2 days of recovery are still able to inhibit SGH10.
**Figure S6:** Gating strategy for immune profiling of colonic lamina propria.

**Table S1:** Summary of LoFreq‐detected variants in the *rmp* and capsule loci.
**Table S2:** Statistical comparison of alpha diversity indices.
**Table S3:** Pairwise PERMDISP analysis of Bray–Curtis dissimilarity.
**Table S4:** List of media components and supplements.
**Table S5:** NUH61 *rmp* locus‐specific primer sequences used for this study.
**Table S6:** Antibodies used for staining colonic lamina propria cells for flow cytometry analysis.

## Data Availability

The data that support the findings of this study are available in the supplementary material of this article. The sequencing datasets generated in this study have been deposited in the NCBI Sequence Read Archive (SRA) under BioProject accession PRJNA1517777 at https://www.ncbi.nlm.nih.gov/bioproject/PRJNA1517777. The data and scripts used are saved in GitHub: https://github.com/Melpy66/Microbiome-analysis. Supplementary materials (methods, figures, tables, graphical abstract, slides, videos, Chinese translated version, and updated materials) may be found in the online DOI or iMeta Science http://www.imeta.science/.
