# Supplementary material for: Different gut microbiome dysbioses impact the evolutionary fate of hypervirulent Klebsiella pneumoniae
Source: Imeta. 2026 Sep 23;5(5):e70175. doi: 10.1002/imt2.70175 (PMC13601767; doi:10.1002/imt2.70175)
Supplement: Supplementary file 1 [file IMT2-5-e70175-s002.docx]

**Supporting information to**

**Different gut microbiome dysbiosis impact the evolutionary fate of hypervirulent *Klebsiella pneumoniae***

**Running title:** Gut microbiome dysbiosis and evolution of hypervirulent *Klebsiella pneumoniae*

Carey Lim^1#^, Jialing Zheng^2#^, Guoxiang Cheam^1#^, Melvin Yong^1^, Hanrong Chen^3^, Andrew Ting^3^, Yahua Chen^1^, Qingqing Wang^2^, Niranjan Nagarajan^1,3^, Yunn-Hwen Gan^1*^

^1^Infectious Diseases Translational Research Programme, Yong Loo Lin School of Medicine, National University of Singapore, Singapore 117545, Republic of Singapore; Department of Biochemistry, Yong Loo Lin School of Medicine, National University of Singapore, Singapore 117596, Republic of Singapore.

^2^Institute of Immunology, Zhejiang University School of Medicine, Hangzhou 310058, and Liangzhu Laboratory, Hangzhou 311121, China.

^3^Genome Institute of Singapore (GIS), Agency for Science, Technology and Research (A*STAR), Singapore 138672, Republic of Singapore.

#These authors contributed equally to the work: Carey Lim, Jialing Zheng, Guoxiang Cheam

*Correspondence: [bchganyh@nus.edu.sg](mailto:bchganyh@nus.edu.sg) (Yunn-Hwen Gan)

**Supplementary methods**

**Bacterial growth media**

Bioreactor media (BRM) and BRM agar are composed of 0.2 % Peptone, 0.2 % Yeast extract, 0.1 % Tryptone, 0.5 % Hemin, 0.015 % Maltose, 0.015 % D-(+)-cellobiose, 0.01 % (+)-Arabinogalactan, 0.02 % Inulin from chicory root, 0.004 % D-(+)-Glucose, Magnesium sulphate, calcium chloride, sodium bicarbonate and potassium phosphate, with or without 1.5 % bacteriological agar. M9 minimal medium was composed of 2 mM magnesium sulfate, 0.1 mM calcium chloride and 0.4 % w/v glucose. The product details of all media and antibiotic supplements are listed in Table S4.

**Bacterial growth assays**

Growth curves were generated by inoculating LB or M9 media supplemented with 20 % glucose by diluting overnight bacterial cultures 2000-fold in 200 µL in a 96-well format and incubating at 37 °C. Bacterial growth was measured by recording OD_600_ in a Tecan Infinite® M Plex microplate every 30 min.

**SGH10 inhibition on solid agar**

Mice were pre-treated with ampicillin or PEG for 5 days, and caeca were harvested and placed immediately in an anaerobic chamber. The caecal contents were removed and diluted in approximately five volumes of pre-reduced PBS. Samples were vortexed and pipetted thoroughly using wide-bore tips to generate a uniform homogenate. All buffers and reagents used in the assay were pre-reduced in the anaerobic chamber for at least 24 h prior to use. For the solid agar co-culture assay, 10^4^ CFU of SGH10 and 25 µL PEG caecal homogenate were spotted onto a cellulose nitrate filter and grown for 24 h in anaerobic conditions. Filters were then vortexed extensively in PBS to release bacteria for SGH10 CFU enumeration on LB agar supplemented with 100 µM carbenicillin. For the spotting assay, SGH10 was streaked onto BRM plates using a cotton swab to grow a bacterial lawn. PEG cecal homogenate was spotted over the lawn, and growth of both SGH10 and PEG cecal communities was observed after 24 h in anaerobic conditions.

**Multiplex cytokine analysis of colon tissue**

Mice were pre-treated with ampicillin or PEG for 5 days, followed by oral gavage inoculation of SGH10. Naïve, post-ampicillin, post-PEG and infected mice at 1 dpi (from both treatment groups) were sacrificed and colons harvested. Faecal matter was removed from the colon, and remaining tissue was homogenised in PBS containing a proteinase inhibitor cocktail (Sigma-Aldrich, PIC0007). Luminex multiplexing assay was then used for simultaneous detection and quantification of multiple analytes in the colon tissue lysates according to the manufacturer’s instructions (ProCartaplex^TM^ Mouse and Rat Mix & Match Panels, Thermo Fisher Scientific). Briefly, samples were incubated with antibody-coated beads in a 96-well plate for 18 h at 4 °C. Following incubation, the bead–analyte complexes were washed and then incubated with biotinylated detection antibodies, followed by streptavidin-phycoerythrin (PE) for signal amplification. After final washes, beads were resuspended in sheath fluid and analysed on the Luminex LX200 system using xPONENT® 4.3 software. A minimum of 20-50 beads per analyte per well was acquired to ensure statistical robustness. Analyte concentrations are extrapolated from standard curves.

**Sequencing of the *rmpA* region of NUH61 non-HMV colonies**

Single colonies of non-HMV mutants of NUH61 were picked from freshly re-streaked LB agar and resuspended in nuclease-free water to serve as templates. Colony PCR was performed using 2X Rapid Taq Master Mix (Vazyme, China) according to the manufacturer’s instructions. Each 25 µL reaction contained 12.5 µL 2X Rapid Taq Master Mix, 1 µL each of forward and reverse primers (10 µM; see Table 2 for primer sequences), 1 uL colony suspension as template, and nuclease-free water to volume. Amplification was carried out on a thermal cycler using the following program: Initial denaturation at 95 °C for 3 min; 33 cycles of 95 °C for 15 s, 50 °C for 15 s, 72 °C for 20 s, followed by a final extension at 72 °C for 5 min. Amplicons were sent for sequencing to Plasmidsaurus (Plasmidsaurus Inc, USA) using their standard amplicon long-read (nanopore-based) sequencing service. Raw reads were aligned to the NUH61 reference genome to detect sequence variation at the *rmp* locus. Primer sequences used can be found in Table S5.

**Variant calling of stool-derived SGH07 bacterial lawn and whole-genome sequencing of isolates**

Ampicillin-treated mice were colonised with 10^6^ CFU of *K. pneumoniae* strain SGH07 by oral gavage. Non-HMV SGH07 colonies were grown in LB, and bacterial pellets were resuspended in 500 µL Zymo 1X DNA/RNA Shield™ before sending to Plasmidsaurus for whole-genome sequencing by Oxford Nanopore Technologies (ONT) sequencing to identify *rmpA* mutations. For variant calling, stools were collected on 5 and 7 dpi, homogenised, and plated on LB agar containing carbenicillin to select for SGH07 lawns, which were scraped for DNA extraction. Sequencing and variant calling were performed as described in the main methods, using the SGH07 genome as the reference.

**DSS-mediated colitis model and whole-genome sequencing of *rmpA* mutants from stool**

Mice were treated with ampicillin for 5 days and then colonised with 10^6^ CFU of SGH10 P*rmpA*-^T^ or *rmpA*^+G^ by oral gavage. At 2 dpi, mice were given sterile 2.5 % dextran sulfate sodium (DSS) in drinking water *ad libitum* for 5 consecutive days before returning them to normal drinking water. On day 8 after starting DSS treatment, stools were collected, homogenised, and plated on LB agar containing carbenicillin to select for SGH10 P*rmpA*-^T^ or *rmpA*^+G^ lawns, which were scraped for DNA extraction. Sequencing and variant calling were performed as described in the main methods, using the genomes of the two mutants as the reference.

**Caecal SCFA quantification**

Short-chain fatty acids (SCFAs) were quantified by GC–MS using an internal standard method. Briefly, ~20 mg of each solid sample was extracted with 500 µL water containing 0.5 % phosphoric acid, homogenised (50 Hz, 3 min), sonicated (10 min), and centrifuged (13,000 × g, 15 min, 4 °C). An aliquot of 200 µL supernatant was mixed with 200 µL n-butanol containing 2-ethylbutyric acid (10 µg/mL) as internal standard, vortexed, sonicated (10 min), and centrifuged (13,000 × g, 5 min, 4 °C). The organic phase was transferred to GC vials for analysis. GC–MS analysis was performed on an Agilent 8890B GC coupled to an Agilent 5977B/7000D MSD (EI, 70 eV) using an HP-FFAP capillary column (30 m × 0.25 mm × 0.25 µm) with helium as carrier gas (1 mL/min). Samples (1 µL) were injected in split mode (10:1) at 230 °C. The oven program was 80 °C, ramp to 120 °C at 40 °C/min, to 180 °C at 10 °C/min, to 200 °C at 20 °C/min, then held at 220 °C for 3 min. The ion source and quadrupole temperatures were 250 °C and 150 °C, respectively, and data were acquired in SIM mode. Quantification of acetic, propanoic, butanoic, isobutyric, valeric, isovaleric, hexanoic, and isohexanoic acids was performed in Agilent MassHunter Quantitative software (v10.0.707.0) using external calibration curves generated from mixed standards (R² > 0.99), with concentrations calculated based on analyte-to-internal standard peak area ratios. Quality control samples were analysed every 5–10 injections, and analytes with RSD < 15 % were considered stable. SCFA content was calculated as (C × V)/M, where C is the measured concentration, V is final extract volume, and M is sample mass. GC–MS analysis was performed on an Agilent 8890B GC coupled to an Agilent 5977B/7000D MSD (EI, 70 eV) using an HP-FFAP capillary column (30 m × 0.25 mm × 0.25 µm) with helium as carrier gas (1 mL/min). Samples (1 µL) were injected in split mode (10:1) at 230 °C. The oven program was 80 °C, ramp to 120 °C at 40 °C/min, to 180 °C at 10°C/min, to 200 °C at 20 °C/min, then held at 220 °C for 3 min. The ion source and quadrupole temperatures were 250 °C and 150 °C, respectively.

**Colonic lamina propria isolation and immune cell profiling**

Tissue harvest and staining procedure can be found in the Methods section of the main text. Gating strategies used to demarcate specific immune cell subtypes for quantification are found in Figure S6. The list of antibodies used for staining can be found in Table S6.

**Supplementary figures**

**Figure S1 Ampicillin and laxative models induce a differential colonic immune response with SGH10 colonisation.** (A) Weight of caecal content harvested from naïve mice or mice treated for 5 days with ampicillin or PEG in drinking water. (B) Hierarchical-clustering heatmaps of Z-scores of immune cell abundances in colonic lamina propria, expressed as percentage of total CD45^+^ cells, comparing naive to ampicillin-treated mice (left) and (C) naive to PEG-treated mice (right) after 5 days of treatment under uninfected conditions (n = 5-9). Changes in each population (% CD45) were analysed by Mann-Whitney test, and p-values are indicated. (D) Colon tissue cytokines were quantified by multiplex bead-based immunoassay. Each point denotes an individual mouse. Data in (A) and (D) are shown as mean ± SD. Data in (A) and (D) were analysed by one-way ANOVA with Tukey’s multiple comparisons test.

**Figure S2 Loss of HMV phenotype in stool bacteria from ampicillin-treated mice. (A)** Representative image of agar plates containing both wild-type and HMV- mutant colonies. Colonies marked with a red “X” were string test negative. (B) L19, L31 and L45 denote individual HMV^-^ colonies obtained from the colon samples of SGH10 colonised mice at 35 dpi. (C) Growth of SGH10, SGH10∆*Kp*VP and NUH61 on LB plates supplemented with carbenicillin alone or with an additional 50 µM tellurite selection. (D) String test results of HMV^+^ and HMV^-^ *Kp*VP^+^ colonies**.** (E) *rmpA* promoter and coding region mutations in NUH61 HMV^-^ colonies collected from colonised mice stools. PCR detection of *Kp*VP plasmid retention in each strain by sampling random HMV^-^ colonies. (F) *rmpA* promoter mutation identified in SGH07 non-HMV isolate by whole-genome sequencing. (G) Mutations identified in the *rmp* locus of SGH07 from stools of colonised mice at days 5 and 7.

**Figure S3 *In vitro* phenotype of SGH10 P*rmpA*^-T^ and *rmpA*^+G^ mutants and *in vivo* reversion in induced colitis model.** (A) Optical density at 600 nm (OD_600_) of culture supernatants following low-speed centrifugation of SGH10, SGH10-GmR and isogenic *wcaJ* or *rmpA* mutants of SGH10. Higher OD_600_ values indicate more mucoviscosity. (B) Growth curve of SGH10, SGH10-GmR and isogenic mutants of *Kp*VP and *rmpA* in LB and M9 supplemented with 0.4 % glucose. Each dot represents one biological repeat. Data are shown as mean ± SD. (C) Poly-T tract reversions observed in colitis mice initially colonised with the *rmpA* promoter mutant (P*rmpA*^-T^). (D) Poly-G tract single nucleotide deletions observed in colitis mice initially colonised with the *rmpA* CDS mutant (*rmpA^+G^*). (E) Amino acid sequence alignment between wild-type SGH10, the *rmpA*^+G^ isogenic mutant and the revertant mutant.

**Figure S4 Distinct microbiome structures under ampicillin and PEG treatment during SGH10 colonisation.** (A) Microbiome composition (relative abundance, %) at the phylum level and (B) at the species level (top 20 species shown per group) for the naïve control, ampicillin-treated groups (Ampicillin_D0, Ampicillin_D5, Ampicillin_D35), and PEG-treated groups (PEG_D0, PEG_D5, PEG_D35).

**Figure S5 Caecal homogenates from PEG-treated mice after 2 days of recovery are still able to inhibit SGH10**. (A) Representative image of caeca from mice treated with 5 days of ampicillin (left) or PEG (right) after 2 days of recovery. (B) Caecal content mass and its (C) total bacterial count from mice after 2 days of recovery. (D) Relative abundance and (E) Linear discriminant analysis Effect Size (LEfSe) identifying differentially abundant bacterial species between ampicillin- and PEG-treated groups after 2 days of recovery with LDA scores indicating effect sizes. (F) Representative images of PEG-treated caecal homogenates mixed with SGH10 and grown on top of filter paper on BRM agar (Left-colonies, right- CFU counts) after 24 h. (G) Representative images of PEG-treated caecal homogenates spotted and grown on a lawn of SGH10 on BRM agar after 24 h. (H) pH of culture medium recorded after 6 h and 24 h of co-incubation of SGH10 with PEG caecal homogenates or filtrates (after 2 days of recovery) buffered with 100 mM MES. (I) Growth of SGH10 in LB or BRM media when acidified to pH 5.5. (J) SCFA quantification in caecal contents collected from naïve, ampicillin- and PEG-treated mice after 2 days of recovery (continued from Figure 6). Each point denotes an individual mouse. Data in (B, C and H) are analysed by unpaired t-test. Data in (F) and (J) are analysed by one-way ANOVA. Data are shown as mean ± SD.

**Figure S6 Gating strategy for immune profiling of colonic lamina propria**. Gating strategy for immune profiling of (A) myeloid lineage cells (B) lymphoid lineage cells (C) and ILC3.
